# Influence of NiTi Wire Diameter on Cyclic and Torsional Fatigue Resistance of Different Heat-Treated Endodontic Instruments

**DOI:** 10.3390/ma15196568

**Published:** 2022-09-22

**Authors:** Eugenio Pedullà, Francesco Saverio Canova, Giusy Rita Maria La Rosa, Alfred Naaman, Franck Diemer, Luigi Generali, Walid Nehme

**Affiliations:** 1 Department of General Surgery and Medical-Surgical Specialties, University of Catania, Via Santa Sofia, 78, 95123 Catania, Italy; 2 Endodontic Department, Saint Joseph University of Beirut, Rue de Damas P.O. Box 17-5208, Beirut 1104 2020, Lebanon; 3 Restorative and Endodontic Department, CHU de Toulouse, University of Toulouse, 31013 Toulouse, France; 4 Clement Ader Institute, UMR CNRS 5312, 31400 Toulouse, France; 5 Endodontic Section, Department of Surgery, Medicine, Dentistry and Morphological Sciences with Transplant Surgery, Oncology and Regenerative Medicine Relevance (CHIMOMO), School of Dentistry, University of Modena and Reggio Emilia, 41125 Modena, Italy

**Keywords:** cyclic fatigue, endodontics, heat treatment, NiTi wire diameter, torsional fatigue, 2Shape

## Abstract

We compared the mechanical properties of 2Shape mini TS2 (Micro-Mega, Besançon, France) obtained from 1.0 diameter nickel-titanium (NiTi) wires and 2Shape TS2 from 1.2 diameter nickel-titanium (NiTi) wires differently thermally treated at room and body temperature. We used 120 NiTi TS2 1.0 and TS2 1.2 files made from controlled memory (CM) wire and T-wire (*n* = 10). Cyclic fatigue resistance was tested by recording the number of cycles to fracture (NCF) at room and body temperatures using a customized testing device. Maximum torque and angle of rotation at failure were recorded, according to ISO 3630-1. Data were analyzed by a two-way ANOVA (*p* < 0.05). The CM-wire files had significantly higher NCFs at both temperatures, independent of wire dimensions. Testing at body temperature negatively affected cyclic fatigue of all files. The 1.0-mm diameter T-wire instruments showed higher NCF than the 1.2-mm diameter, whereas no significant differences emerged between the two CM wires at either temperature. The maximum torque was not significantly different across files. The TS2 CM-wire files showed significantly higher angular rotation to fracture than T-wire files. The TS2 CM-wire prototypes showed higher cyclic fatigue resistance than T-wire prototypes, regardless of wire size, exhibiting suitable torsional properties. Torsional behavior appears to not be affected by NiTi wire size.

## 1. Introduction

Since endodontic instruments have been manufactured with nickel-titanium [NiTi] wire, the use of NiTi files has largely increased in endodontics [1]. NiTi endodontic instruments possess extreme flexibility and strength, even if they are vulnerable to fracture during use in clinical situations [2]. The main causes leading to fracture of NiTi files are torsional and cyclic fatigue [3]. Fracture caused by torsional fatigue occurs when the file engages the root canals as the handpiece continues to rotate. Torsional failure is defined by a maximum torsional load and angle of rotation. This last property is connected to the file’s ability to twist before fracture [2]. Cyclic fatigue occurs when the file rotates freely within the canal, and is exposed to repeated cycles of compression and traction in the part of the root canal with the greatest curvature [3]. Although both failure modes simultaneously occur in clinical situations, many studies have found that cyclic fatigue fracture is the principal cause of file separation [4].

Several factors, including file size, cross-sectional area, design, heat treatment, and metallurgical properties of the instruments, affect the mechanical properties of rotary files [5]. Recent studies have also shown that temperature is another factor influencing the mechanical behavior of NiTi instruments. A significant decrease in the cyclic fatigue strength of the instruments was found when the files were tested at body temperature [6,7,8].

In this context, heat treatments have been proposed to prevent the fracture of NiTi files and improve the metallurgical properties of these instruments, modifying the austenite finish temperature [9]. Above this temperature, instruments exist completely as austenitic structures that results in more rigidity than martensitic ones [10,11]. Some examples of heat treatment technology include controlled memory wire (CM; Coltene, Cuyahoga Falls, OH, USA) [12] and T-wire (Micro-Mega, Besançon, France). CM-wire heat treatment is a distinctive process that modifies the memory of the material [13], whereas T-wire treatment, according to the manufacturer, improves flexibility and cyclic fatigue resistance [14]. One of the file systems constituted from T-wire alloy is 2Shape (TS) (Micro-Mega) [1]. The TS file system possesses a triple-helix cross-section and is produced in two sizes: TS1 (size #25/0.04) and TS2 (size #25/0.06) [5]. In addition, TS2 files can be produced from a 1.0-mm or 1.2-mm diameter NiTi wire, which generates TS2 mini (1.0) and TS2 (1.2) files, respectively. Both TS2 mini 1.0 and TS2 1.2 files have the same tip and taper (25.06) but differ in the amount of metal due to the different diameters of the NiTi wire used. To date, no studies have compared the cyclic fatigue resistance of NiTi TS2 mini 1.0 and TS2 1.2 rotary files or T-wire and CM-wire 2Shape TS2 files, because the latter ones are available as prototypes.

Therefore, this study aims to evaluate and compare the cyclic and torsional fatigue resistance of differently heat-treated (i.e., T-wire and CM-wire) TS2 mini 1.0 and TS2 1.2 files. The null hypotheses were: (i) the wire diameter does not affect fatigue resistance of tested instruments at room and body temperature; (ii) the wire diameter does not interfere in the maximum torque load or angular rotation of instruments with different heat treatments.

## 2. Materials and Methods

No human or animal subjects were used in this study; therefore, ethics committee approval was not required [15]. Based on the results of similar previous studies [2,4], sample size estimation was calculated a priori with G*Power 3.1.9.2 software (Heinrich-Heine- University at Dusseldorf, Dusseldorf, Germany) to have 80% power and alpha error probability of 0.05.

The 2Shape TS2 mini 1.0-mm and TS2 1.2-mm files, differently heat-treated (i.e., T-wire and prototypes CM-wire), were used. All files were 25 mm long, with 10 instruments in each group used in cyclic fatigue and torsional resistance tests. Prior to testing, all files were inspected using a stereomicroscope (SZR- 10; Optika, Bergamo, Italy) to verify any deformation [2]. None were discarded.

### 2.1. Cyclic Fatigue Test

**Figure 1:**
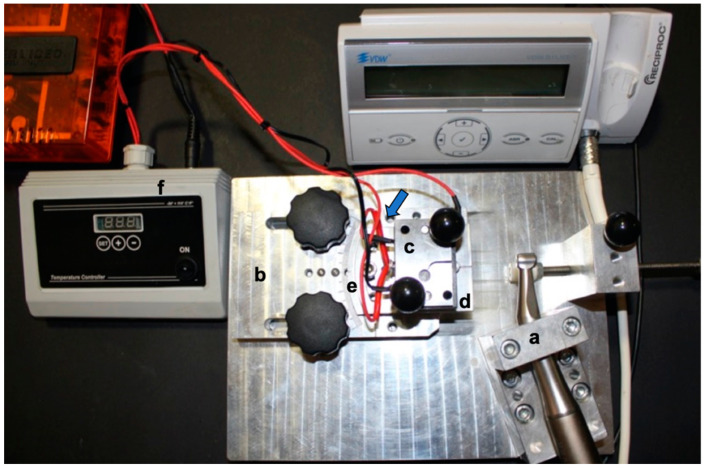
An illustrative figure of the customized testing device employed for cyclic fatigue tests. (a) The electric handpiece was maintained in a stable position by a block system; (b) a mobile support on rails allowed the insertion/withdrawal of the NiTi file in a standardized manner in the 0.06 tapered artificial canal (c); (d) a mobile platform permitted to put the file in different inclinations marked by the angles reported on the mobile support (all files were tested at 0°) (e); (f) a thermostat was used to check the temperature with an acceptable variation of ±1 °C. The blue arrow indicates the thermocouple applied to the artificial canal.

**Figure 2:**
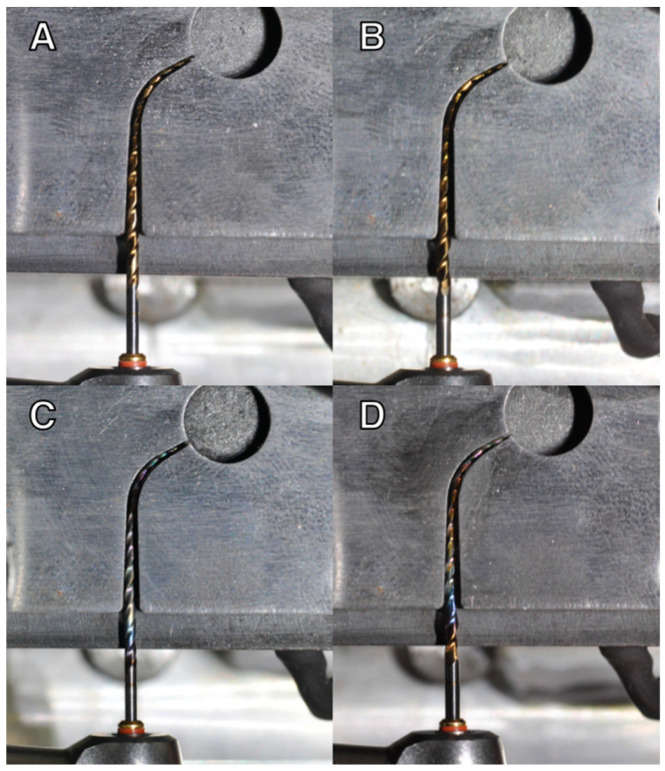
(**A**) TS2 T-wire 1.0, (**B**) 1.2, and (**C**) CM-wire 1.0, (**D**) 1.2 in the artificial canal with 60° angle of curvature and 5-mm radius at body temperature.

Twenty instruments from each of these systems were tested in vitro with a static cyclic fatigue test at room (25 ± 1 °C) and body (37 ± 1 °C) temperatures, for a total of 80 NiTi rotary instruments. Cyclic fatigue testing was performed using a custom machine (Figure 1) with a stationary unit that maintained a 6:1 electric reduction handpiece (Sirona Dental Systems GmbH, Bensheim, Germania) in a fixed three-dimensional position and a movable rail mount that allowed file positioning within the artificial canal. All instruments were placed in a precise and reproducible manner using the electric handpiece [5] (Figure 2).

**Figure 3:**
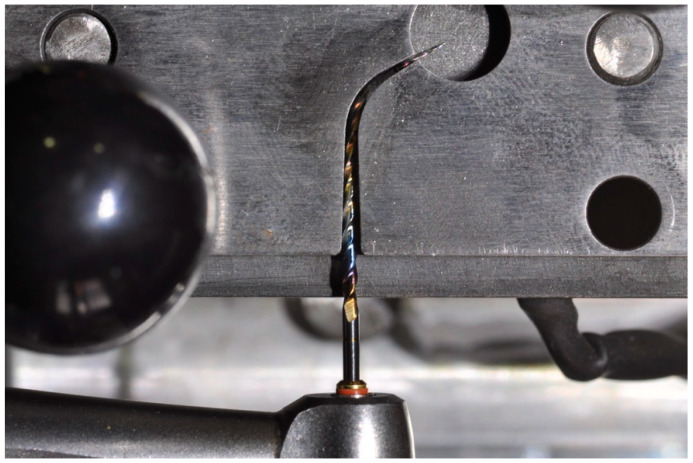
Part of instrument using TS2 CM-wire 1.2 that fractured during the cyclic fatigue test at body temperature.

The mobile platform contained a 16 mm-long stainless steel artificial canal, with an angle of 60 degrees and a radius of 5 mm curvature [15]. The artificial canal was specifically designed for the TS2 instrument employed in terms of size (25) and taper (0.06), giving it a sufficient lumen with suitable trajectory. For a better clinical simulation [16,17], the temperature adjustment was guaranteed by a thermostat which ensured the proper heating of the artificial canal containing the instrument. The temperature was maintained constantly during the test by means of a thermocouple applied to the artificial canal, which activated or deactivated the thermostatic resistance when the temperature decreased or reached the preset one, respectively. The TS2 mini 1.0 and TS2 1.2 were tested using a continuous rotation at 300 rpm (revolutions per minute) and at the maximum torque (4.1 Ncm) until they broke [5] (Figure 3). A special high-flow synthetic oil (Super Oil; Singer Co Ltd., Elizabeth, NJ) was applied as a lubricant. The time to fracture was recorded with a stopwatch and confirmed by video, recorded using a digital camera [18]. The number of cycles to fracture (NCF) was also calculated using the equation “rpm/60 × time to fracture (seconds)” [18].

### 2.2. Torsional Fatigue Test

For the torsional test, each instrument was clamped at 3 mm from the tip by a chuck connected to a torque-sensing load cell; after this, the shaft of the file was fastened into an opposing chuck, able to be rotated with a stepper motor in the clockwise direction at a speed of 2 revolutions per minute until file breakage. The torque load (Ncm) and angular rotation (°) were monitored continuously by a torsiometer (Sabri Dental Enterprises, Downers Grove, IL, USA) at room temperature (21 °C ± 1 °C), and the ultimate torsional strength and angle of rotation at failure were recorded.

### 2.3. SEM Analysis

The fracture surfaces of all fragments were observed under a scanning electron microscope (SEM) (ZEISS Supra 35VP; GmBH, Oberkochen, Germany) for topographic features of the fractured instruments.

### 2.4. Statistical Analysis

Once the normality of distributions and equality of variances were confirmed by the Shapiro–Wilk and Levene tests, respectively, statistical analysis was performed using a two-way analysis of variance and Tukey’s post hoc multiple comparison test, with NiTi wire dimension and heat treatment as the independent variables. The significance level was set at 5% (*p* < 0.05) using the statistical software Prism 8.0 (GraphPad Software, Inc., La Jolla, CA, USA).

## 3. Results

The means and standard deviations of cyclic fatigue resistance, torque maximum load, and angle of rotation until fracture for each instrument are shown in Table 1.

The TS2 CM-wire prototype files had the statistically highest cyclic fatigue resistance, at both temperatures, independently from the wire dimensions (*p* < 0.05).

When comparing T-wire files, TS2 mini 1.0 exhibited higher cyclic fatigue resistance than TS2 1.2 at both temperatures (*p* < 0.05). With regard to CM-wire files, no significant differences emerged between the two wire dimensions at room or body temperature (*p* < 0.05). Finally, being at body temperature reduced the fracture resistance of all instruments (*p* < 0.05).

The mean length of the fractured fragment (5 ± 0.2 mm) was not significantly different for the instruments tested (*p* < 0.05). No significant difference emerged in the maximum torque values between the tested instruments (*p* < 0.05). The CM-wire TS2 files showed significantly higher angular rotation to fracture than TS2 T-wire instruments (*p* < 0.05), with no significant difference between the two wire dimensions for both heat-treatments.

**Figure 4:**
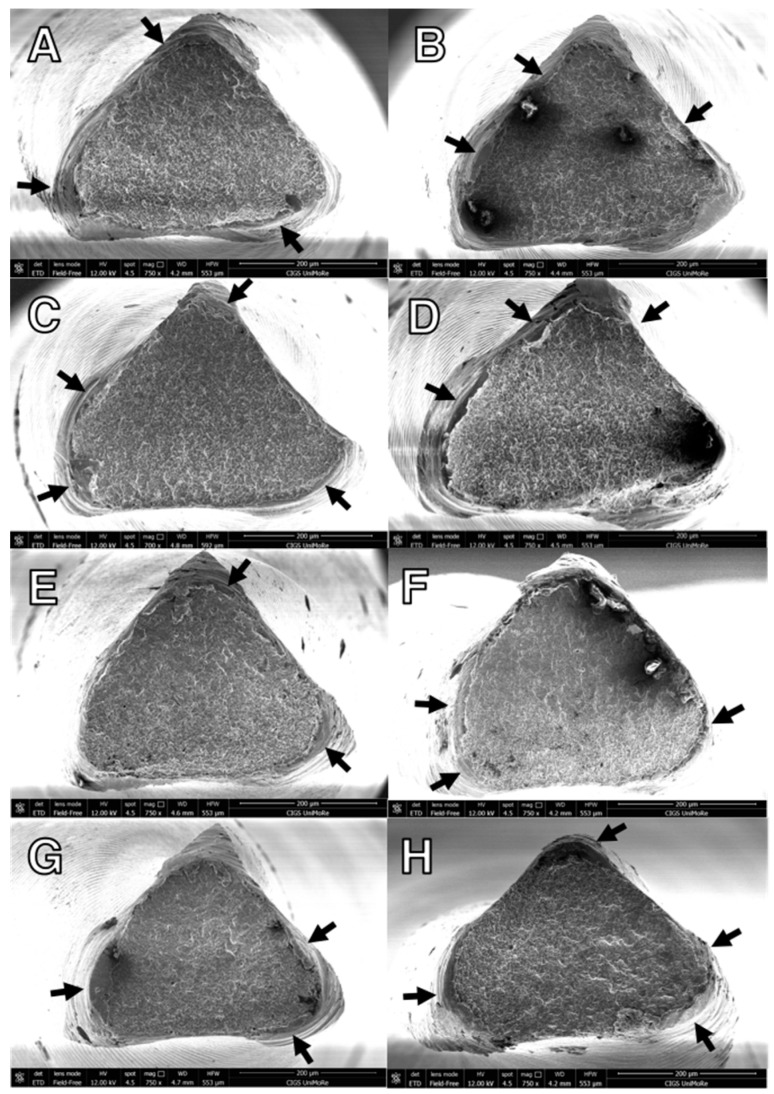
Representative field-emission scanning electron microscope images of the instruments after fatigue tests: (**A**,**B**) TS2 mini 1.0 T-wire, (**C**,**D**) TS2 1.2 T-wire, (**E**,**F**) TS2 mini 1.0 CM-wire, and (**G**,**H**) TS2 1.2 CM-wire, at 25 °C ± 1 °C (**A**,**C**,**E**,**G**) and 37 °C ± 1 °C (**B**,**D**,**F**,**H**). Typical features of cyclic fatigue fracture of the ductile fatigue area with microdimples and cones are observed with the black arrows indicating the origins of crack initiation.

Scanning electron microscopy of the fracture surfaces exhibited similar and typical patterns of cyclic fatigue for all tested instruments. The crack initiation area and overload fast fracture zone for cyclic fatigue failure are shown in Figure 4.

## 4. Discussion

The current study aimed to assess the influence of NiTi wire dimensions on the mechanical properties of differently heat-treated TS2 prototypes. Although natural teeth best represent clinical conditions, variation in root canal anatomy is difficult to standardize in laboratory conditions [1]. Therefore, a stainless-steel canal, as previously described [15], was used for cyclic fatigue tests [15]. Moreover, the customized device ensured the reproducibility of the tests [15,16,17]. The correct placement of each file into the artificial canal was confirmed by the similar lengths of the fractured segments from the tested instruments [9]. Torsional tests were performed following the ISO Standard 3630-1, as previously reported [19].

According to the results of the present study, wire dimension had no significant effect for TS2 prototypes produced with CM heat treatment, which were significantly more resistant than TS2 prototypes with T-wire heat treatment, at both temperatures, independent of NiTi wire dimension. Conversely, the TS2 mini 1.0 T-wire showed higher cyclic fatigue resistance than the TS2 1.2 T-wire, at both temperatures. Thus, the first null hypothesis could be partially rejected. The results obtained for CM-wire files could be attributed to the heat treatment itself, which confers the rotary files more flexibility, as previously reported [3,20], compensating for the difference in the diameter dimensions of the NiTi wire of the two instruments [21,22]. The advantage provided by the T-wire heat treatment is less than the CM-wire [5,20,23], and consequently, the benefit of the smaller amount of metal in the TS2 mini 1.0 files is significantly more pronounced in the T-wire files in terms of increased flexibility and resistance to cyclic fatigue [3].

Moreover, our results showed that body temperature reduced the fracture resistance of all the instruments tested. These results support previous studies that found a reduction in the cyclic fatigue resistance of NiTi rotary instruments exposed at body temperature, due to changes in crystalline phases induced by the temperature increase [24,25,26].

Torsional tests showed that wire dimension had no significant effect on either maximum torque or angle of rotation to fracture. Thus, the second null hypothesis could not be rejected. The CM files exhibited significantly higher values of angular rotation than the T-wire files, whereas no significant difference emerged in terms of maximum torque values. The higher angular rotation (a parameter associated with ductile fractures) [19,27] could be attributable to the major flexibility of the CM-treatment [28], which does not impair maximum torque as confirmed by the similar values obtained with T-Wire files. In addition, the lack of a significant difference between the two wire dimensions suggest that torsional behavior is probably affected more by heat-treatment than by wire diameter. Since no authors have evaluated the mechanical properties of TS2 instruments with different NiTi wire diameters, some of our results cannot be confirmed by different studies.

SEM analysis revealed that fracture surfaces exhibited similar typical patterns of cyclic fatigue for all tested instruments. Of note, fractographic analysis of 1.0 T-wire files revealed a wider area covered by fatigue striations than in 1.2 T-wire instruments, thus suggesting slower fracture propagation after surface microcracks formed and, therefore, a higher resistance [29]. This analysis further corroborates the improvement in cyclic fatigue resistance of 2Shape 1.0 T-wire instruments when compared with their 1.2 counterparts.

Although the customized device we used was able to control all laboratory variables, we still faced some limitations. The artificial canal was specifically designed for the TS2 instrument employed, in terms of size (25) and taper (0.06), to ensure standardized conditions for both 0.06 tapered files. Yet, differences in file dimension could influence the adaptation of the file into the artificial walls and should be considered for future research. In clinical practice, many other factors, such as operator experience and anatomical complexities, act simultaneously, affecting the mechanical behavior of files used [30,31]. In addition, differences in the mechanical behavior of tested instruments should be determined, considering multiple factors including the phase transformation temperatures of heat-treated alloys [32]. CM alloys have been extensively investigated [28], whereas no data are available on T-wire phase transformation temperature. Future studies should address this matter. Furthermore, all variables associated with different NiTi endodontic files, such as design and dimensions, could impact their fracture resistance and need to be explored.

A previous study investigated the influence of environmental temperature on the torsional behavior of 25.06 conventional NiTi alloy and CM thermal-treated NiTi instruments, reporting no significant differences between files tested at 21 and 35 °C [33]. Thus, the authors hypothesized that variations in temperature would not affect torsional behavior. However, the differences in methodological conditions and lack of other findings require further investigations to confirm this hypothesis.

Within these limits, the present study has relevant clinical implications: heat treatments combined with a different NiTi wire dimensions (and in turn, different amounts of metal) could significantly affect the mechanical behavior of files. Limited to these laboratory conditions: CM-wires exhibited higher flexibility independently of wire dimensions, maintaining suitable torsional behavior; the reduction in NiTi wire dimension from 1.2 (TS2) to 1.0 (TS2 mini) increased fatigue resistance for T-wire heat-treated TS2. Clinicians should choose flexible instruments when flexural stress is high, such as in accentuate curvatures.

Future studies should investigate how the effects of NiTi wire dimensions combined with different heat treatments and file designs affect the mechanical properties of NiTi instruments. 

## 5. Conclusions

Within the limits of the present study, the TS2 prototypes made by controlled-memory alloy showed higher cyclic fatigue resistance than conventional TS2 T-wires, independent of wire dimensions and test temperatures, exhibiting suitable torsional properties. In addition, the 1.0 wire diameter positively affected the cyclic fatigue resistance of TS2 mini T-wires, at both tested temperatures, whereas it had no effect on torsional behavior of CM-wire or T-wire heat-treated instruments.

## Figures and Tables

**Table 1:** Number of cycles to fracture [NCF] at room and body temperature, maximum torque [Ncm], and angle of rotation until fracture [°] values [mean ± standard deviation] of the different heat-treated TS2 instruments.

	Number of Cycles to Fracture [NCF]		Torque [Ncm]	Angle of Rotation [°]
	25 °C ± 1 °C	37 °C ± 1 °C		
Instrument	Mean ± SD	Mean ± SD	Mean ± SD	Mean ± SD
**TS2 1.0 T-wire**	395 ^a1^ ± 50	244 ^b1^ ± 42	0.41 ^1^ ± 0.06	294 ^1^ ± 42
**TS2 1.2 T-wire**	217 ^a2^ ± 48	142 ^b2^ ± 26	0.38 ^1^ ± 0.05	271 ^1^ ± 19
**TS2 1.0 CM-wire**	2153 ^a3^ ± 391	1608 ^b3^ ± 204	0.45 ^1^ ± 0.05	423 ^2^ ± 43
**TS2 1.2 CM-wire**	2303 ^a3^ ± 420	1825 ^b3^ ± 316	0.40 ^1^ ± 0.08	505 ^2^ ± 87

Same letters show differences not statistically significant between instruments in the same row (*p* < 0.05). Same numbers show differences not statistically significant between instruments in the same column (*p* < 0.05).

## Data Availability

The data presented in this study are available on request from the corresponding author.
